# Themes in Superhero-Based Television Shows: An Opportunity for the Development of Children and Adolescents Through Co-Viewing and Active Mediation

**DOI:** 10.7759/cureus.7965

**Published:** 2020-05-05

**Authors:** Raymond Y Kim, Morgan K Moroi, Amalia Brawley, Marian Poley, Tonya S King, Robert Olympia

**Affiliations:** 1 Orthopedics, Penn State College of Medicine, Hershey, USA; 2 Cardiac/Thoracic/Vascular Surgery, Penn State College of Medicine, Penn State Milton S. Hershey Medical Center, Hershey, USA; 3 Obstetrics and Gynecology, Penn State College of Medicine, Penn State Milton S. Hershey Medical Center, Hershey, USA; 4 Internal Medicine and Pediatrics, University of Maryland, Baltimore, USA; 5 Public Health Sciences, Penn State College of Medicine, Hershey, USA; 6 Emergency Medicine and Pediatrics, Penn State Hershey Medical Center, Hershey, USA

**Keywords:** superhero, television, media, violence, co-viewing, active mediation

## Abstract

The depiction of superheroes and comic book characters in television shows has become incredibly popular. The objective of this study was to determine the positive and negative themes depicted in a select number of superhero-based television shows. A total of 49 episodes from 10 superhero-based television shows were analyzed by four independent reviewers. The average number of positive and negative themes was 18.8 and 36.9 mean events per hour, respectively, for all included episodes. The most common positive themes in our sample were associated with service, teamwork, and encouragement, and the most common negative themes were associated with violence, bullying, and alcohol use. Although exposure to positive themes depicted in superhero-based television shows may be beneficial to the development of children and adolescents, pediatric health care providers should counsel families in an attempt to limit their exposure to violence and other negative themes depicted in this genre of television shows.

## Introduction

With on-demand Internet video streaming services like Netflix and Hulu becoming vogue, superhero-based television shows have become more accessible to viewers of all ages. Superheroes are often viewed by children as altruistic role models who make a positive impact on society. Nonetheless, superhero-based television shows often depict negative themes, such as violence, conflict, and substance use, which may influence risk-taking behaviors and choices made by children and adolescents. Repeated exposure to violence in media, whether in television shows, films, video games, or music, may pose a significant risk to the emotional and social development of children and adolescents. Published studies have shown that media violence may attribute to aggressive behavior, desensitization to violence, and nightmares in children [1-2]. Such findings have led the American Academy of Pediatrics to issue a formal statement on media violence, encouraging pediatricians to advocate for a safer media environment and to counsel patients and their parents to limit exposure to violence [3].

In 1996, the broadcasting industry created a voluntary ratings system named TV Parental Guidelines (http://www.tvguidelines.org), intended to accompany all television programming. In response to public concerns regarding increasingly explicit sexual content, graphic violence, and strong profanity in television programs, the rating system was established to alert parents to the content and age-appropriateness of television programming. Rating categories include the following: TV-Y (appropriate for all ages, especially children aged two to six years), TV-Y7 (appropriate for children aged seven years and above), TV-G (suitable for all ages; containing little or no violence, no strong language, and little or no sexual dialogue or situations), TV-PG (parental guidance suggested; may contain one or more of the following: some suggestive dialogue, infrequent coarse language, sexual situations, or moderate violence), TV-14 (parents strongly cautioned; unsuitable for children under the age of 14 years, may contain one or more of the following: intensely suggestive dialogue, strong coarse language, intense sexual situations, or intense violence), and TV-MA (mature audience only, unsuitable for children under age 17 years; containing one or more of the following: crude indecent language, explicit sexual activity, graphic violence). Unfortunately, studies have demonstrated that TV Parental Guidelines may be ineffective in discriminating risk behaviors, such as violence, sexual behavior, alcohol use, and smoking, in television shows [4-5]. As a consequence, children and adolescents may unknowingly be exposed to excess violence and other risk-taking behaviors while watching television shows that their parents believe to be age-appropriate.

A recently published study examined the positive and negative themes depicted in superhero-based films [6]. Based on their sample of 30 superhero-based films, the authors determined that the most common positive themes were “assisting others/protecting the public,” “positive relationships with family/friends,” and “teamwork/collaboration." The most common negative themes were “acts of violence/fighting,” “use of guns/knives/lethal weapons”, and “bullying/intimidation/torture”. There have been no published studies analyzing themes depicted in superhero-based television shows. The objective of this study was to determine the positive and negative themes depicted in a select number of superhero-based television shows. The authors hope that by identifying positive and negative themes found in superhero-based television shows, pediatric healthcare providers and parents will use this research to develop co-viewing strategies to help in the education and development of children and adolescents who watch this genre.

## Materials and methods

We conducted a content analysis study examining the positive and negative themes depicted in 10 different superhero television shows. The 10 television shows included in the analysis were selected from a list published on a popular television and film website, www.imdb.com, searching for “TV series/ TV mini-series, superhero (sorted by popularity ascending)” released between 2012 and 2017. For television shows that aired over multiple seasons, we analyzed the first season of each show. We used a random number generator to select which episodes to analyze for every show and analyzed at least 25% of episodes contained in every show included in the study.

A data collection instrument, developed by the authors and composed of a predetermined list of positive and negative themes (Table 1), allowed each of the four viewers (RYK, MKM, AMB, MP) to record each event associated with a theme depicted in each selected episode. Events, either positive or negative, were defined as actions, discussions, or references stated directly in the television script or implied during a scene. Actions performed in the television episode and then later referenced were coded only at the initial encounter. Certain coding guidelines were determined prior to viewing the episodes to standardize the data collection process. For example, when coding an extended fight sequence, a contained battle was coded as a single event (“Act of violence/fighting”) while each use of a weapon (“Use of guns/knives/lethal weapons”) was coded individually per event. Further, when coding a sequence involving several superheroes that are working together to protect the public, the entire sequence was coded as one event (“Helping/assisting others and mankind/protecting the public”) while each interaction between superheroes during the sequence was coded as an individual event (“Teamwork/collaboration/cooperation”).

**Table 1:** List of positive and negative themes included in the data collection instrument

Positive Themes
Helping/assisting others and mankind/protecting the public
Importance of intelligence over force and power (clever, wit)
Honesty
Forgiveness/not holding a grudge
Overcoming adversity/obstacles/hardships
Self-sacrifice
Learning from past mistakes
Justice/Karma – getting what one deserves
Justice – triumph of law enforcement
Taking responsibility for actions/apologizing
Following the rules / Doing the right thing
Teamwork/collaboration/cooperation
Mentorship
Encouraging/Compliments
Preparation and setting goals
Combatting racism/prejudice
Self-defense
Negative Themes
Bullying / intimidation / torture / threat
Murder – Single (1 victim)
Murder – Multiple (1-4 victims)
Murder – Mass-casualty (> 4 victims)
Arguing / verbal altercation
Excessive anger/emotion
Acts of violence / fighting
Use of guns / knives / lethal weapons (depiction with usage)
Exposure to guns / knives / lethal weapons (depiction without usage)
Property destruction
Invincibility / desire for world dominance
Importance of force and power over intelligence
Taking advantage of the weak
Cheating / lying / manipulating
Planning revenge / holding a grudge
Not following the rules / Doing the wrong thing / breaking the law
Selfishness
Succumbing to peer pressure
Placing blame on others
Discouragement / Putting someone down
Betrayal towards colleagues, family, friends
Theft / stealing
Drinking alcohol
Smoking cigarettes
Drug use
Sexual activity
Objectification of women
Racism or prejudice

Each of the four reviewers watched and coded every episode included in the study independently. Data collection instrument sheets were collected by the primary investigator (RYK) and entered into Microsoft Excel (Microsoft Corporation, Redmond, Washington). The rates of total positive and negative themes per hour were then estimated using repeated measures Poisson regression models in SAS version 9.4 (SAS Institute, Cary, North Carolina) with adjustment for the reviewer, and reported with corresponding 95% confidence intervals. The rates of individual positive and negative themes were estimated in the same manner. 

The institutional review board at the Pennsylvania State Hershey Medical Center deemed the study exempt.

## Results

A total of 49 episodes from our sample of 10 superhero-based television shows were analyzed. Table 2 describes the characteristics of the television shows that were included in the study.

**Table 2:** Superhero-based television show descriptions * General television show information from imdb.com **PG (parental guidance suggested), TV-14 (parents strongly cautioned), MA (mature audience only) ***Dominant themes were those with the highest number of events per hour

Title	Year released	Rating**	Average length of episode (minutes)	Network	Dominant positive theme ***	Dominant negative theme ***
Agents of S.H.I.E.L.D.	2013	PG	45	ABC	Teamwork	Exposure to guns/knives/lethal weapons
Arrow	2012	TV-14	42	The CW	Helping Others	Exposure to guns/knives/lethal weapons
Daredevil	2015	MA	54	ABC	Helping Others	Use of guns/knives/lethal weapons
Flash	2014	PG	43	The CW	Teamwork	Violence/Fighting
Gotham	2014	TV-14	42	FOX	Helping Others	Violence/Fighting
Iron Fist	2017	MA	55	Netflix	Teamwork	Violence/Fighting
Jessica Jones	2015	MA	56	Netflix	Helping Others	Violence/Fighting
Legends of Tomorrow	2016	TV-14	42	The CW	Teamwork	Violence/Fighting
Luke Cage	2016	MA	55	Netflix	Helping Others	Exposure to guns/knives/lethal weapons
Supergirl	2015	PG	43	The CW	Helping Others	Bullying/Torture

The average number of positive and negatives themes was 18.8 (95% CI: 16.9 - 20.9) and 36.9 (95% CI: 33.9 - 40.2) mean events per hour for all included shows, respectively (with adjustment for reviewer variability p<0.001). Table 3 and Table 4 show the most common positive and negative themes depicted in our sample of superhero-based television shows, respectively.

**Table 3:** Most common positive themes (reported as mean events per hour [95% confidence interval]) depicted in our sample of superhero-based television episodes* *Themes with mean events per hour under 0.5 for all included television episodes include: triumph of law enforcement (0.43 [0.26 – 0.72]), self-sacrifice (0.42 [0.28 – 0.60]), overcoming adversity/obstacles/hardships (0.36 [0.23 – 0.56]), mentorship (0.31 [0.17 – 0.56]), and learning from past mistakes (0.20 [0.12 – 0.32]). Themes with mean events per hour too small to estimate for all included television episodes include: following the rules / doing the right thing, combatting racism/prejudice, and self-defense.

Positive themes	All included episodes
Helping/assisting others and mankind/protecting the public	3.59 [3.17 – 4.08]
Teamwork/collaboration/cooperation	3.51 [3.04 – 4.06]
Encouraging/Compliments	2.38 [1.94 – 2.92]
Taking responsibility for actions/apologizing	1.57 [1.23 – 1.96]
Honesty	1.41 [1.12 – 1.78]
Importance of intelligence over force and power (clever, wit)	0.85 [0.63 – 1.14]
Preparation and setting goals	0.64 [0.47 – 0.86]
Justice/Karma – getting what one deserves	0.56 [0.37 – 0.85]
Forgiveness/not holding a grudge	0.52 [0.36 – 0.74]

**Table 4:** Most common negative themes (reported as mean events per hour [95% confidence interval]) depicted in our sample of superhero-based television episodes* *Themes with mean events per hour under 0.5 for all included television episodes include: not following the rules / doing the wrong thing / breaking the law (0.48 [0.34 – 0.68]), taking advantage of the weak (0.33 [0.22 – 0.49]), sexual activity (0.33 [0.20 – 0.53]), succumbing to peer pressure (0.29 [0.19 – 0.44]), drug use (0.27 [0.12 – 0.61]), invincibility / desire for world dominance (0.23 [0.12 – 0.42]), objectification of women (0.20 [0.10 – 0.39]), placing blame on others (0.19 [0.12 – 0.31]), importance of force and power over intelligence (0.17 [0.09 – 0.33], selfishness (0.16 [0.08 – 0.32]), and smoking cigarettes (0.03 [0.01 – 0.09]) Themes with mean events per hour too small to estimate for all included television episodes include: murder – mass-casualty (> 4 victims) and racism or prejudice.

Negative themes	All included episodes
Acts of violence / fighting	5.49 [4.91 – 6.14]
Exposure to guns / knives / lethal weapons	4.74 [4.09 – 5.50]
Use of guns / knives / lethal weapons	4.53 [4.02 – 5.11]
Bullying / intimidation / torture / threat	3.85 [3.32 – 4.47]
Arguing / verbal altercation	3.01 [2.55 – 3.56]
Murder – Single (1 victim)	1.75 [1.39 – 2.20]
Property destruction	1.54 [1.23 – 1.93]
Drinking alcohol	1.36 [1.10 – 1.69]
Excessive anger/emotion	1.32 [1.02 – 1.70]
Cheating / lying / manipulating	1.26 [1.02 – 1.57]
Discouragement / Putting someone down	1.01 [0.69 – 1.48]
Planning revenge / holding a grudge	0.88 [0.65 – 1.17]
Betrayal towards colleagues, family, friends	0.81 [0.63 – 1.05]
Murder – Multiple (1-4 victims)	0.63 [0.44 – 0.92]
Theft / stealing	0.53 [0.34 – 0.80]

Based on our sample of superhero-based television shows,Agents of S.H.I.E.L.D, Daredevil, and Iron Fisthad the highest overall positive theme mean events per hour (Table 5) while Flash, Arrow, and Iron Fist had the highest overall negative theme mean events per hour (Table 6).

**Table 5:** Total number of positive themes (mean events per hour) per television show season *PG (parental guidance suggested), TV-14 (parents strongly cautioned), MA (mature audience only)

Title	Rating *	Mean events per hour
Agents of S.H.I.E.L.D.	PG	27.70
Daredevil	MA	25.87
Iron Fist	MA	24.08
Arrow	TV-14	19.55
Gotham	TV-14	19.03
Jessica Jones	MA	18.06
Legends of Tomorrow	TV-14	16.55
Flash	PG	14.85
Luke Cage	MA	12.47
Supergirl	PG	12.06

**Table 6:** Total number of negative themes (mean events per hour) per television show season *PG (parental guidance suggested), TV-14 (parents strongly cautioned), MA (mature audience only)

Title	Rating *	Mean events per hour
Flash	PG	54.55
Arrow	TV-14	43.98
Iron Fist	MA	40.37
Jessica Jones	MA	37.17
Agents of S.H.I.E.L.D.	PG	36.83
Luke Cage	MA	36.22
Daredevil	MA	34.02
Legends of Tomorrow	TV-14	30.36
Supergirl	PG	27.64
Gotham	TV-14	27.15

The repeated measures Poisson regression models for the total number of both positive and negative themes per hour found a statistically significant amount of inter-rater variability among the reviewers (p<0.001 in each of the models).

## Discussion

From the introduction of Superman, which marked the rise of the Golden Ages of Comic Books in 1938, to today, when Marvel’s Avengers: Endgame shattered the all-time global box-office opening record, superheroes have always been a vital aspect of American culture [7-8]. In the beginning, the original Superman was far different from the Superman we are familiar with today. Superhuman powers including flight, X-ray-vision, and wind control, are later developments that have been added throughout the years. In the early years, Superman did not fight the supervillains with the extreme violence that we have grown accustomed to today but rather fought the real villains of the New Deal era while standing up for the common people. The early villains consisted of bosses who failed to provide safe working environments, stockbrokers selling fraudulent stocks, and congressmen conspiring with corrupt corporations. It is no coincidence that the birth of the comic book era paralleled the Great Depression era and the struggles Americans experienced during that time.

In stark contrast, Superman of today can be seen in the film Man of Steel violently ending his epic battle against his adversary, General Zod by gruesomely snapping his neck, which seemed reflective of the darker and more violent nature of superhero cinema in the modern era. Research presented at the 2018 American Academy of Pediatrics National Conference and Exhibition reported that, contrary to the popular belief, protagonists (“good guys”) performed more acts of violence in their sample of superhero-based films compared with antagonists (“bad guys”) [9]. An increase in violence is not only true for superhero-based films. The violence depicted in films has doubled since the 1950s, and gun violence in PG-13 rated films has more than tripled since 1985 [10]. Further, in a review of 74 G-rated animated films, every film in their study contained at least one act of violence with the majority (55%) of the violence being associated with good or neutral characters utilizing a wide range of weapons during their violent acts [11]. Lastly, in a recently published study analyzing positive and negative themes depicted in 45 top-grossing films released between 2005 and 2015, the most common negative themes were associated with violence [12].

Based on our sample of superhero-based television shows, there were approximately twice as many negative themes as positive themes depicted. The most common positive themes were associated with service, teamwork, and encouragement. The most common negative themes were associated with violence, bullying, and alcohol use. These themes are similar to those depicted in a published study analyzing themes depicted in superhero-based films. There was a 60% overlap of the 10 most common positive themes and a 70% overlap of the 10 most common negative themes when comparing superhero-based films and television shows [6]. Common positive themes found in our sample of superhero-based television shows, and not in their sample of superhero-based films, included “honesty,” “justice/karma - getting what one deserves,” and “forgiveness/not holding a grudge.” Common negative themes found in our sample of superhero-based television shows, and not in their sample of superhero-based films, included “property destruction,” “drinking alcohol” and “discouragement/putting someone down.”

Given the significant amount of time children dedicate to media consumption, evidence has shown that visual media, such as television and film, contribute to a child’s psychosocial development, both positively and negatively. The most common positive themes in our sample of superhero-based television shows were associated with service, teamwork, and encouragement. Children who are frequently exposed to positive themes in television shows may learn important life lessons from characters’ situations and then translate these lessons into their own lives. Studies have shown that the content of visual media may help children develop positive thoughts and behaviors, such as sharing, improved social interactions, racial attitudes, cooperation, and empathy, and influence the perspective of gender-based roles in our society [13-14].

The most common negative themes in our sample of superhero-based television shows were associated with violence, bullying, and alcohol use. Several published studies show that media violence exposure may lead to the development of non-ideal behaviors such as aggression, bullying, antisocial attitudes, and other forms of violence [3,15-16]. The rate of bullying and cyberbullying has significantly increased over the past 10 years [17]. Studies have demonstrated that exposure to antisocial media content may be related to an increase in bullying and cyberbullying performed by their pediatric viewing audience [18-19]. Lastly, although our sample of superhero-based television shows demonstrate adults engaging in socially appropriate behavior with alcohol, depicting the intake of alcohol may glorify such behavior in the eyes of children and adolescents. Alcohol exposure in films has shown to be a predictor for the earlier initiation of heavy episodic drinking and sexual debut [20].

The TV Parental Guidelines were developed in response to public concerns regarding increasingly explicit sexual content, graphic violence, and strong profanity in television programs. Unfortunately, many parents believe the television rating system to be challenging; 68% of the parents of 10 to 17 years old reported that they do not use the television rating system at all [21]. Further, parents find the rating system to be highly inaccurate, with objective parental evaluation showing 50% of television shows rated TV-14 to be inappropriate for their adolescent children [5]. Based on our sample of superhero-based television shows, we found inaccuracies in the content of violence. Both Flash and Arrow, rated PG and TV-14, respectively, depicted the highest negative theme mean-events per hour, compared with other shows in our sample that were rated MA. We suggest that television shows be assigned a rating based on both the severity of violence (moderate, intense, or graphic) and the number of violent events depicted per episode.

To help families prevent the harmful effects of unhealthy media exposure, the American Academy of Pediatrics recommends asking two media-related questions at every well-child visit: “How much recreational screen time does your child or teenager consume daily?” and “Is there a TV set or an Internet-connected electronic device in their bedroom [22]?" When children and adolescents are identified as significant viewers of visual media, clinicians should further evaluate for aggressive behaviors, fears, or sleep disturbances and intervene if deemed necessary.

Despite the availability of parental control settings available on modern televisions and hand-held devices to restrict younger viewers from age-inappropriate content, it is unrealistic to believe that children and adolescents will not be exposed to superhero-related television shows due to the availability of these shows on video-sharing online websites or via streaming on television or mobile electronic devices. It is important for pediatric health care providers and parents to be aware of the content of superhero-related television shows and to understand the impact that positive and negative themes have on the children and adolescents who view them. A method to optimize the development of children and adolescents who view superhero-related television shows involves co-viewing these shows as a family and active mediation. Parents play an active role in discussing both positive and negative themes depicted in the visual media to help their children think critically about distinguishing between right and wrong. Previous research has shown that active mediation was related to decreased levels of aggression, sexual behavior, and substance use in children exposed to age-inappropriate content [23]. We recommend the use of a template for co-viewing proposed by Bauer et al. (“HEROES/VILLAINS”), based on the most common positive and negative themes depicted in a sample of superhero-based films, along with a template developed by the authors (“TVSHOW” (Table 7)), based on positive and negative themes we found to be prevalent in our sample of superhero-based television shows.

**Table 7:** Template for co-viewing superhero-based television shows We encourage families to use the template below (“TVSHOW,” focusing on superhero-based television shows), with the “HEROES/VILLAINS” template (based on themes depicted in a sample of superhero-based films) 6, either during or after viewing the television episode, to provoke thoughtful conversation and reflection by having the child answer each of the questions based on common positive and negative themes found in superhero-based television shows.

T	Truthfulness / honesty	Identify a character in the television show that demonstrates truthfulness and/or honesty. Why is it important to be honest? When was the last time that you were honest with someone? How did it make you feel?
V	Vindication / justice /getting what one deserves	Give an example of justice being served in a good way. What are the proper ways that you can attain justice?
S	Swallowing alcohol	Give an example of a character in the television show who uses or abuses alcohol? Were there any consequences to the character’s use of alcohol? Do you drink alcohol? Have you dealt with any consequences to drinking alcohol? Have you ever been pressured to drink alcohol? How did you respond to this pressure?
H	Harbor no grudge against / forgiveness	Identify a character in the television show that forgives another character. Have you demonstrated forgiveness recently? How did it make you feel?
O	Obstruct one’s hopes / discourage / putting someone down	Identify a character in the television show that discourages another character. Has someone recently put you down or discouraged you to follow your dreams? How did you respond to this discouragement?
W	Wrecking property	Identify a character in the television show that has destroyed someone’s property. Have you ever destroyed someone else’s property? Why did you do this? How could you have rectified the situation?

There are several limitations to our study. Since the television shows included in our sample were selected based on subjective reports of the “most popular” superhero-based television shows released between 2012 and 2017 as reported by one popular online site (www.imdb.com), our choices may not represent the most popular or most-watched shows by children and adolescents or be generalizable to television shows outside our release date range. As certain shows included in our study require a paid-subscription to services such as Netflix, it may be possible that certain children and adolescents may be inadvertently excluded from watching these shows. Another limitation of our study was the limited sampling of the episodes. As we randomly selected a set number of episodes to analyze for each show, we extrapolated the content of positive and negative themes based on our randomized sample of episodes and, therefore, our data may not represent the entire season for each included superhero-based television show.

The coding of the television show episodes also represents a limitation. We found a substantial inter-viewer variability between the reviewers for both positive and negative themes. Although coding guidelines were decided prior to viewing the study episodes, each reviewer may have interpreted scenarios and dialogue associated with positive and negative themes differently, depending on variables such as background, gender, race, and age of the reviewer. Furthermore, all the reviewers were adults, who may interpret themed events differently than children and adolescents. The reviewers were also all in the medical profession who were familiar with the presence of violence in media, which may have contributed to our own personal bias while watching the films. Although our objective was to determine the positive and negative themes depicted in a sample of superhero-based television shows, the actual number of events may not be as important as the positive and negative themes that are represented frequently or under-represented and require more emphasis in future television shows.

Although our primary goal was to determine the thematic content of superhero-based television shows in our sample, we did not determine the positive and negative effects that exposure to these themes have on their pediatric audiences. 

## Conclusions

Based on our sample of shows, there were approximately twice as many negative themes as positive themes depicted. Although exposure to positive themes found in superhero-based television shows may be beneficial to the development of children, pediatric health care providers should counsel children and their families in the attempt to limit their exposure to violence and other negative themes depicted in this genre of television shows. We encourage co-viewing and active mediation, focusing on depicted positive and negative themes, as a method to guide children and adolescents through critical periods of their mental and behavioral development.
